# Supplementary material for: Meta-Analysis and Gene Set Enrichment Relative to ER Status Reveal Elevated Activity of MYC and E2F in the “Basal” Breast Cancer Subgroup
Source: PLoS One. 2009 Mar 9;4(3):e4710. doi: 10.1371/journal.pone.0004710 (PMC2650420; doi:10.1371/journal.pone.0004710)
Supplement: Dataset S1 — GSEA results from first screen. The file contains a zipped archive of the HTML results of the second GSEA screen. Reports for enriched or depleted datasets may be accessed from the “index.html” link within the my_analysis.GseaPreranked.1219975950625.rpt.zip ZIP archive. Enrichment plots are available for each of the 50 most enriched and depleted gene sets. (13.32 MB ZIP) [file pone.0004710.s001.zip › my_analysis.GseaPreranked.1219975950625.rpt.zip!//HOFFMANN_BIVSBII_BI_TABLE2.html]

Details for gene set HOFFMANN\_BIVSBII\_BI\_TABLE2[GSEA]

|  || Dataset | zSco.ERneg.to.ERpos\_collapsed |
| Phenotype | NoPhenotypeAvailable |
| Upregulated in class | na\_pos |
| GeneSet | HOFFMANN\_BIVSBII\_BI\_TABLE2 |
| Enrichment Score (ES) | 0.5611149 |
| Normalized Enrichment Score (NES) | 2.502312 |
| Nominal p-value | 0.0 |
| FDR q-value | 0.0 |
| FWER p-Value | 0.0 |
Table: GSEA Results Summary

  

Fig 1: Enrichment plot: HOFFMANN\_BIVSBII\_BI\_TABLE2      
 Profile of the Running ES Score & Positions of GeneSet Members on the Rank Ordered List

  

| PROBE | GENE SYMBOL | GENE\_TITLE | RANK IN GENE LIST | RANK METRIC SCORE | RUNNING ES | CORE ENRICHMENT || 1 | CDC20 |  |  | 9 | 7.151 | 0.0142 | Yes |
| 2 | TTK |  |  | 11 | 7.059 | 0.0288 | Yes |
| 3 | CCNE1 |  |  | 24 | 6.716 | 0.0418 | Yes |
| 4 | BUB1 |  |  | 41 | 6.445 | 0.0540 | Yes |
| 5 | CENPA |  |  | 51 | 6.315 | 0.0664 | Yes |
| 6 | CCNA2 |  |  | 52 | 6.297 | 0.0795 | Yes |
| 7 | NASP |  |  | 59 | 6.201 | 0.0919 | Yes |
| 8 | AURKB |  |  | 76 | 6.038 | 0.1033 | Yes |
| 9 | ANP32E |  |  | 84 | 5.988 | 0.1152 | Yes |
| 10 | TRIP13 |  |  | 96 | 5.890 | 0.1266 | Yes |
| 11 | CDC45L |  |  | 108 | 5.824 | 0.1379 | Yes |
| 12 | KIF2C |  |  | 124 | 5.703 | 0.1486 | Yes |
| 13 | RAD54L |  |  | 146 | 5.602 | 0.1586 | Yes |
| 14 | KIFC1 |  |  | 156 | 5.525 | 0.1694 | Yes |
| 15 | DBF4 |  |  | 159 | 5.516 | 0.1807 | Yes |
| 16 | RFC3 |  |  | 164 | 5.493 | 0.1918 | Yes |
| 17 | MELK |  |  | 181 | 5.424 | 0.2019 | Yes |
| 18 | MCM6 |  |  | 198 | 5.317 | 0.2117 | Yes |
| 19 | MCM10 |  |  | 201 | 5.315 | 0.2226 | Yes |
| 20 | MTHFD2 |  |  | 205 | 5.307 | 0.2334 | Yes |
| 21 | CKS2 |  |  | 239 | 5.145 | 0.2416 | Yes |
| 22 | KIF4A |  |  | 266 | 5.031 | 0.2501 | Yes |
| 23 | STMN1 |  |  | 310 | 4.907 | 0.2570 | Yes |
| 24 | KIF20A |  |  | 318 | 4.874 | 0.2666 | Yes |
| 25 | CDCA3 |  |  | 321 | 4.868 | 0.2765 | Yes |
| 26 | ANP32B |  |  | 329 | 4.860 | 0.2861 | Yes |
| 27 | NCAPH |  |  | 348 | 4.799 | 0.2947 | Yes |
| 28 | CKS1B |  |  | 416 | 4.638 | 0.2993 | Yes |
| 29 | CCNB2 |  |  | 431 | 4.589 | 0.3077 | Yes |
| 30 | SMC4 |  |  | 448 | 4.519 | 0.3159 | Yes |
| 31 | NT5DC2 |  |  | 453 | 4.499 | 0.3249 | Yes |
| 32 | AURKA |  |  | 460 | 4.483 | 0.3338 | Yes |
| 33 | CHAF1B |  |  | 479 | 4.411 | 0.3416 | Yes |
| 34 | STIM1 |  |  | 499 | 4.352 | 0.3492 | Yes |
| 35 | RAD51 |  |  | 508 | 4.332 | 0.3576 | Yes |
| 36 | DLG7 |  |  | 521 | 4.306 | 0.3656 | Yes |
| 37 | PLK4 |  |  | 524 | 4.292 | 0.3744 | Yes |
| 38 | PRC1 |  |  | 543 | 4.253 | 0.3819 | Yes |
| 39 | TUBA1 |  |  | 563 | 4.214 | 0.3892 | Yes |
| 40 | TUBB |  |  | 582 | 4.183 | 0.3965 | Yes |
| 41 | PSAT1 |  |  | 621 | 4.087 | 0.4021 | Yes |
| 42 | SMC2 |  |  | 651 | 4.028 | 0.4083 | Yes |
| 43 | CCDC99 |  |  | 737 | 3.863 | 0.4098 | Yes |
| 44 | FEN1 |  |  | 754 | 3.838 | 0.4166 | Yes |
| 45 | TMPO |  |  | 792 | 3.788 | 0.4216 | Yes |
| 46 | CENPE |  |  | 808 | 3.766 | 0.4283 | Yes |
| 47 | NUSAP1 |  |  | 836 | 3.732 | 0.4340 | Yes |
| 48 | RANBP5 |  |  | 838 | 3.729 | 0.4417 | Yes |
| 49 | HSPD1 |  |  | 869 | 3.673 | 0.4470 | Yes |
| 50 | SRF |  |  | 889 | 3.644 | 0.4532 | Yes |
| 51 | H2AFX |  |  | 930 | 3.591 | 0.4576 | Yes |
| 52 | UBE2C |  |  | 994 | 3.489 | 0.4600 | Yes |
| 53 | KIF23 |  |  | 1030 | 3.442 | 0.4645 | Yes |
| 54 | SSX2IP |  |  | 1038 | 3.427 | 0.4711 | Yes |
| 55 | TUBB3 |  |  | 1109 | 3.350 | 0.4728 | Yes |
| 56 | E2F8 |  |  | 1122 | 3.334 | 0.4788 | Yes |
| 57 | BRCA2 |  |  | 1126 | 3.331 | 0.4855 | Yes |
| 58 | HMGB3 |  |  | 1128 | 3.330 | 0.4923 | Yes |
| 59 | DNA2L |  |  | 1190 | 3.249 | 0.4944 | Yes |
| 60 | MDN1 |  |  | 1209 | 3.227 | 0.4998 | Yes |
| 61 | VPS72 |  |  | 1274 | 3.164 | 0.5015 | Yes |
| 62 | COL4A3 |  |  | 1335 | 3.098 | 0.5033 | Yes |
| 63 | ACTN4 |  |  | 1357 | 3.070 | 0.5081 | Yes |
| 64 | DDX19A |  |  | 1363 | 3.067 | 0.5141 | Yes |
| 65 | HELLS |  |  | 1382 | 3.057 | 0.5191 | Yes |
| 66 | CIT |  |  | 1464 | 2.983 | 0.5191 | Yes |
| 67 | WDHD1 |  |  | 1576 | 2.872 | 0.5166 | Yes |
| 68 | HMMR |  |  | 1608 | 2.843 | 0.5202 | Yes |
| 69 | CCNB1 |  |  | 1626 | 2.823 | 0.5248 | Yes |
| 70 | RELN |  |  | 1673 | 2.780 | 0.5270 | Yes |
| 71 | LDHA |  |  | 1699 | 2.761 | 0.5309 | Yes |
| 72 | NEK2 |  |  | 1703 | 2.758 | 0.5364 | Yes |
| 73 | BIRC3 |  |  | 1726 | 2.745 | 0.5404 | Yes |
| 74 | CKAP5 |  |  | 1738 | 2.731 | 0.5452 | Yes |
| 75 | CDC25C |  |  | 1794 | 2.700 | 0.5467 | Yes |
| 76 | TUBA6 |  |  | 1802 | 2.689 | 0.5517 | Yes |
| 77 | XPO1 |  |  | 1805 | 2.687 | 0.5572 | Yes |
| 78 | CDKN3 |  |  | 1827 | 2.674 | 0.5611 | Yes |
| 79 | HCK |  |  | 1992 | 2.537 | 0.5539 | No |
| 80 | ECT2 |  |  | 2026 | 2.509 | 0.5566 | No |
| 81 | RRM2 |  |  | 2047 | 2.485 | 0.5602 | No |
| 82 | KIN |  |  | 2241 | 2.358 | 0.5505 | No |
| 83 | SREBF2 |  |  | 2252 | 2.349 | 0.5546 | No |
| 84 | RPA1 |  |  | 2380 | 2.270 | 0.5496 | No |
| 85 | HES1 |  |  | 2503 | 2.198 | 0.5449 | No |
| 86 | CBX5 |  |  | 2621 | 2.133 | 0.5404 | No |
| 87 | TK1 |  |  | 2730 | 2.076 | 0.5365 | No |
| 88 | GALT |  |  | 2752 | 2.066 | 0.5392 | No |
| 89 | DNMT1 |  |  | 2844 | 2.017 | 0.5365 | No |
| 90 | PHF17 |  |  | 3017 | 1.924 | 0.5274 | No |
| 91 | ITGB7 |  |  | 3076 | 1.896 | 0.5269 | No |
| 92 | PRM1 |  |  | 3138 | 1.871 | 0.5262 | No |
| 93 | CDKN2C |  |  | 3148 | 1.866 | 0.5294 | No |
| 94 | EIF2AK2 |  |  | 3438 | 1.735 | 0.5110 | No |
| 95 | GDPD5 |  |  | 3524 | 1.698 | 0.5080 | No |
| 96 | CLCN2 |  |  | 3527 | 1.695 | 0.5114 | No |
| 97 | TOP2A |  |  | 3735 | 1.610 | 0.4990 | No |
| 98 | TUBB2C |  |  | 3736 | 1.610 | 0.5023 | No |
| 99 | TBXA2R |  |  | 3943 | 1.538 | 0.4898 | No |
| 100 | POLA1 |  |  | 3967 | 1.525 | 0.4913 | No |
| 101 | RFC5 |  |  | 4034 | 1.500 | 0.4894 | No |
| 102 | RACGAP1 |  |  | 4097 | 1.475 | 0.4877 | No |
| 103 | ABCA1 |  |  | 4273 | 1.403 | 0.4773 | No |
| 104 | NUDC |  |  | 4541 | 1.306 | 0.4597 | No |
| 105 | LIG1 |  |  | 4659 | 1.266 | 0.4534 | No |
| 106 | SPINK1 |  |  | 4757 | 1.235 | 0.4486 | No |
| 107 | RRM1 |  |  | 4872 | 1.197 | 0.4424 | No |
| 108 | MMP14 |  |  | 5099 | 1.116 | 0.4275 | No |
| 109 | PIM2 |  |  | 5147 | 1.099 | 0.4262 | No |
| 110 | SAMHD1 |  |  | 5458 | 0.990 | 0.4047 | No |
| 111 | EFNB3 |  |  | 5674 | 0.923 | 0.3902 | No |
| 112 | RBP4 |  |  | 5827 | 0.870 | 0.3805 | No |
| 113 | INCENP |  |  | 5907 | 0.845 | 0.3762 | No |
| 114 | IDUA |  |  | 6130 | 0.775 | 0.3609 | No |
| 115 | GOT1 |  |  | 6419 | 0.677 | 0.3404 | No |
| 116 | AARS |  |  | 6422 | 0.675 | 0.3417 | No |
| 117 | EDN2 |  |  | 6495 | 0.652 | 0.3375 | No |
| 118 | FBP2 |  |  | 6498 | 0.650 | 0.3387 | No |
| 119 | NUCKS1 |  |  | 6663 | 0.602 | 0.3275 | No |
| 120 | TRIM46 |  |  | 6668 | 0.600 | 0.3284 | No |
| 121 | CD55 |  |  | 6686 | 0.593 | 0.3284 | No |
| 122 | LSP1 |  |  | 6706 | 0.584 | 0.3282 | No |
| 123 | PSMC3 |  |  | 6733 | 0.577 | 0.3274 | No |
| 124 | RPL10 |  |  | 6800 | 0.551 | 0.3235 | No |
| 125 | CD2 |  |  | 6802 | 0.549 | 0.3246 | No |
| 126 | ACTN2 |  |  | 7137 | 0.440 | 0.3000 | No |
| 127 | AZIN1 |  |  | 7475 | 0.319 | 0.2751 | No |
| 128 | ATP5D |  |  | 7543 | 0.291 | 0.2706 | No |
| 129 | IL4 |  |  | 7605 | 0.268 | 0.2665 | No |
| 130 | PTPRS |  |  | 7672 | 0.239 | 0.2619 | No |
| 131 | FHL1 |  |  | 7929 | 0.136 | 0.2427 | No |
| 132 | BAZ2A |  |  | 7968 | 0.117 | 0.2401 | No |
| 133 | MYH11 |  |  | 7989 | 0.109 | 0.2388 | No |
| 134 | PPIA |  |  | 8142 | 0.059 | 0.2274 | No |
| 135 | IDE |  |  | 8326 | -0.006 | 0.2134 | No |
| 136 | PSEN2 |  |  | 8361 | -0.021 | 0.2109 | No |
| 137 | ANP32A |  |  | 8388 | -0.031 | 0.2090 | No |
| 138 | LIPC |  |  | 8400 | -0.039 | 0.2082 | No |
| 139 | ANK3 |  |  | 8438 | -0.052 | 0.2055 | No |
| 140 | RAG1 |  |  | 8450 | -0.056 | 0.2048 | No |
| 141 | PPP2CA |  |  | 8612 | -0.134 | 0.1928 | No |
| 142 | WT1 |  |  | 8652 | -0.153 | 0.1902 | No |
| 143 | TUBA3 |  |  | 8675 | -0.160 | 0.1888 | No |
| 144 | MYEF2 |  |  | 8677 | -0.160 | 0.1891 | No |
| 145 | KIF22 |  |  | 8725 | -0.180 | 0.1859 | No |
| 146 | SPIN |  |  | 8778 | -0.200 | 0.1823 | No |
| 147 | GJA4 |  |  | 8970 | -0.300 | 0.1684 | No |
| 148 | NELF |  |  | 9127 | -0.373 | 0.1573 | No |
| 149 | CBX1 |  |  | 9417 | -0.522 | 0.1364 | No |
| 150 | AATK |  |  | 9547 | -0.588 | 0.1278 | No |
| 151 | IL4R |  |  | 9609 | -0.623 | 0.1245 | No |
| 152 | BCL3 |  |  | 9660 | -0.650 | 0.1220 | No |
| 153 | HOXC5 |  |  | 9738 | -0.690 | 0.1176 | No |
| 154 | GSN |  |  | 9824 | -0.739 | 0.1126 | No |
| 155 | GSPT1 |  |  | 9841 | -0.751 | 0.1130 | No |
| 156 | HNRPA0 |  |  | 9885 | -0.775 | 0.1113 | No |
| 157 | CASP3 |  |  | 9899 | -0.783 | 0.1120 | No |
| 158 | ABR |  |  | 9931 | -0.798 | 0.1113 | No |
| 159 | CAPN1 |  |  | 9938 | -0.802 | 0.1125 | No |
| 160 | COIL |  |  | 9942 | -0.805 | 0.1139 | No |
| 161 | CAPG |  |  | 10021 | -0.858 | 0.1098 | No |
| 162 | JUN |  |  | 10156 | -0.934 | 0.1015 | No |
| 163 | RAB4B |  |  | 10524 | -1.188 | 0.0760 | No |
| 164 | SLC29A1 |  |  | 10768 | -1.365 | 0.0604 | No |
| 165 | CDKN1A |  |  | 10787 | -1.380 | 0.0619 | No |
| 166 | SCN1B |  |  | 10954 | -1.533 | 0.0524 | No |
| 167 | HSD11B2 |  |  | 11245 | -1.780 | 0.0340 | No |
| 168 | CAMP |  |  | 11384 | -1.917 | 0.0275 | No |
| 169 | DBNDD2 |  |  | 11547 | -2.085 | 0.0195 | No |
| 170 | RAMP1 |  |  | 11680 | -2.202 | 0.0140 | No |
| 171 | GPHN |  |  | 11980 | -2.540 | -0.0034 | No |
| 172 | ARL1 |  |  | 12003 | -2.563 | 0.0002 | No |
| 173 | EVL |  |  | 12166 | -2.798 | -0.0063 | No |
| 174 | NOP17 |  |  | 12300 | -2.965 | -0.0103 | No |
| 175 | IER3 |  |  | 12423 | -3.140 | -0.0130 | No |
| 176 | NEDD4 |  |  | 12585 | -3.407 | -0.0182 | No |
| 177 | PCK2 |  |  | 12617 | -3.462 | -0.0134 | No |
| 178 | PPP1CA |  |  | 12748 | -3.737 | -0.0155 | No |
| 179 | CD151 |  |  | 12752 | -3.747 | -0.0079 | No |
| 180 | SEPP1 |  |  | 12828 | -3.949 | -0.0054 | No |
| 181 | PRRG2 |  |  | 12961 | -4.367 | -0.0064 | No |
| 182 | RABEP1 |  |  | 13106 | -4.853 | -0.0073 | No |
| 183 | CRIP2 |  |  | 13180 | -5.244 | -0.0019 | No |
| 184 | HSPB8 |  |  | 13256 | -6.029 | 0.0049 | No |
Table: GSEA details [plain text format]

  

Fig 2: HOFFMANN\_BIVSBII\_BI\_TABLE2: Random ES distribution      
 Gene set null distribution of ES for **HOFFMANN\_BIVSBII\_BI\_TABLE2**
